# Association between SQSTM1 dysregulation and risk in alopecia areata: a Mendelian randomization study

**DOI:** 10.3389/fimmu.2025.1652444

**Published:** 2025-11-25

**Authors:** Lifang Hu, Sheng Wan, Xiuzu Song

**Affiliations:** Department of Dermatology, Hangzhou Third People’s Hospital, Hangzhou, China

**Keywords:** alopecia areata, autoimmunity, autophagy, inflammation, metabolic reprogramming, SQSTM1

## Abstract

**Background:**

Alopecia areata (AA) is an autoimmune disease typified by nonscarring hair loss. It manifests as a heterogeneous disorder with diverse clinical presentations and variable treatment responses, underscoring the significance of identifying novel biomarkers for precision management.

**Objective:**

This study aims to explore the relationship between metabolic reprogramming-related genes (MRRGs) and the risk of developing AA.

**Methods:**

MRRGs were identified through the GeneCards database and existing literature. Genetic instruments were obtained from the eQTLGen database, and AA-related data were retrieved from the OpenGWAS database. The TwoSampleMR R package was applied for statistical analysis. Additionally, RT-qPCR and immunofluorescence assays were performed to validate the expression of target genes in AA-affected hair follicles and healthy controls.

**Results:**

Six MRRGs (DLD, NFE2L2, SDHB, SLC2A1, PSAT1, and SQSTM1) showed significant causal associations with AA. RT-qPCR analysis revealed markedly elevated SQSTM1 mRNA levels in AA-affected hair follicles compared with healthy controls. Immunofluorescence confirmed increased SQSTM1 protein accumulation alongside reduced LC3B-II expression in AA-affected hair follicles.

**Conclusions:**

This study underscores the significant association between SQSTM1 and AA, advancing our understanding of AA pathophysiology.

## 1 Introduction

AA is a common chronic tissue-specific autoimmune disease, resulting in hair loss, that affects up to 2% of the general population and is thought to arise from a multifactorial interplay of genetic, environmental, and immunological factors (1). This condition not only affects the physical appearance of individuals but also has profound implications for their psychological well-being and overall quality of life (2). Current pharmacological treatments primarily include corticosteroids, Janus kinase inhibitors (JAKi), and other immunosuppressants. However, a significant subset of patients experiences suboptimal responses and frequent relapses, highlighting the pressing need for further research to elucidate the underlying mechanisms of AA and identify novel therapeutic targets (3).

Recent advancements in the field of metabolic reprogramming have begun to shed light on its significant role in various diseases, including cancers and metabolic disorders. Metabolic reprogramming refers to the shifts in cellular metabolism that occur in response to pathological conditions, enabling cells to adapt and thrive in their altered environments (4, 5). However, the specific relationship between metabolic reprogramming and AA remains largely unexplored, creating a significant research gap that warrants further investigation. Our study employs Mendelian randomization analysis (6) for identifying potential biomarkers and therapeutic targets that could enhance the management of AA.

## 2 Materials and methods

This study was conducted and reported in accordance with the STROBE-MR (Strengthening the Reporting of Observational Studies in Epidemiology - Mendelian Randomization) guidelines (7).

### 2.1 Genes involved in metabolic reprogramming

First of all, we used the GeneCards database (8) (https://www.genecards.org/) to collect MRRGs, which provides comprehensive information on human genes. Afterwards, by keeping only MRRGs with “Protein Coding” and “Relevance Score > 3”, 94 MRRGs were obtained. Subsequently, we collected the MRRGs from references (9) through the PubMed, and a total of 5 MRRGs were obtained. Finally, the MRRGs from the two sources were merged, and a total of 96 MRRGs were obtained.

### 2.2 cis-eQTL dataset

We downloaded the cis-eQTL dataset related to metabolic reprogramming from eQTLGen (https://www.eqtlgen.org). The eQTLGen dataset is based on 31,684 peripheral blood and peripheral blood mononuclear cell (PBMC) samples, which were derived from multiple large European population cohorts (predominantly individuals of European ancestry). The primary objective of this dataset is to identify associations between genetic variations and gene expression in blood tissue.

The eQTL analysis was performed following a standardized and unified pipeline, with covariates such as age, sex, principal components, and technical batches adjusted for in the model. We downloaded the complete set of statistically significant cis-eQTL results after multiple-testing correction (FDR < 0.05), and selected single nucleotide polymorphisms (SNPs) significantly associated with gene expression (*P value* < 5×10^-8^) as genetic instrumental variables (IVs). Additionally, we obtained allele frequencies from eQTLGen, which were calculated using allele counts reported by all participating cohorts.

### 2.3 Outcome data set

We obtained the genome-wide association study (GWAS) data for the outcome variable alopecia areata (AA) (GWAS ID: finn-b-L12_ALOPECAREATA) from the MRC IEU OpenGWAS database (10). This dataset is derived from the 12th release of the FinnGen study, and standardized association summary statistics were retrieved as the outcome using the R package TwoSampleMR (11).

The GWAS data for alopecia areata (AA) comprises 289 AA cases and 211,139 control samples, all of which were sourced from European populations. The GWAS analysis was based on genome-wide genotyping data, and a linear mixed model was employed to control for potential confounding factors including sex, age, and genetic principal components. The results provide effect estimates, standard errors, and *P value* with reference to the effect allele.

Since both this outcome data and the exposure data (from eQTLGen) are based on European populations, with independent samples and consistent population background, this can effectively reduce population stratification bias and satisfy the assumptions of Mendelian randomization analysis.

### 2.4 Selection of instrumental variables

A valid genetic variation IVs must satisfy three core assumptions: firstly, the hypothesis of association, that is, the selected IVs must be significantly related to the exposure factor. Secondly, independence assumption, that is, the IVs must not be significantly related to potential confounders that might affect the exposure or outcome. Thirdly, exclusivity limitation, that is, the IVs could only affect the outcome through the path of “instrumental variable → exposure → outcome”. must not exert an indirect effect on the outcome through other pathways.

The screening criteria for SNPS of IVs in this study were as follows: *P<*5×10–^8^ of SNPS in the exposure GWAS was used as the screening criteria. Linkage Disequilibrium (LD) screening was performed to ensure the independence of the selected SNPs; Only SNPS that were not in linkage disequilibrium (SNPS with r<0.001 ^2^and a physical distance of >10000 kb between every two genes) were included. Subsequently, the corresponding genetic effect values were extracted from the outcome GWAS based on the screened SNPs. F-statistics were calculated to assess weak instrumental variable bias. We selected the IVs with F>10 for subsequent analyses, because F<10 indicates that the genetic variant used is a weak instrumental variable, which might bias the results to some extent (12). The formula for calculating the F-statistic is as follows:


$$F=\frac{N-k-1}{k}\times \frac{R^{2}}{1-R^{2}}$$

Where n is the sample size, k is the number of instrumental variables used, and R^2^ reflects the extent to which the instrumental variables explain the exposure. R^2^ = 2× (1-MAF) ×MAF×^2^β, where MAF is the minimum allele frequency and β is the allele effect size. In addition, SNPS with MAF>0.01 were selected to exclude bias caused by rare variants.

### 2.5 Univariate MR analysis

We performed a two-sample MR analysis using the TwoSampleMR package with MRRGs obtained in eQTLGen as the exposure factor and AA as the outcome. We used the Wald ratio method to evaluate the results of MR for exposures containing only one SNP, and IVW method to evaluate the results of MR for exposures containing two or more SNPS. In the absence of pleiotropy and with or without heterogeneity, the IVW and Wald ratio methods were used as the main MR Methods, supplemented by the other four methods (causal combinations with heterogeneity were excluded). When pleiotropy was present, then MR-Egger method was used to calculate the results. Finally, Steiger’s directivity test was used to determine whether the direction of causality was correct.

### 2.6 Sensitivity analysis

Sensitivity analysis was carried out to ensure the reliability of the results the results in the following ways by using various methods such as heterogeneity test, pleiotropy test and one-by-one exclusion test, as follows:

Firstly, heterogeneity test: The Cochran Q test was used to evaluate the heterogeneity among the SNP estimates. If the Cochran Q test was statistically significant, the analysis results were proved to be significantly heterogeneous. The random effects model of IVW was used to estimate the causal effect size for highly heterogeneous results. Because the Cochran Q test could only test the presence or absence of heterogeneity, it could not test the distribution of heterogeneity. Therefore, the I^2^ statistic was used to reflect the proportion of heterogeneous part of IVs in the total variation: when I^2^ ≤ 0, it was set to 0, indicating that no heterogeneity was observed. I^2^ = 0-25%, indicating mild heterogeneity; I^2^ = 25%-50%, indicating moderate heterogeneity; I^2^>50% indicated high heterogeneity. The specific calculation formula is as follows:


$$I2=\frac{Q-df}{Q}\times 100\%$$

Secondly, pleiotropy test: MR-Egger method was used to test the pleiotropy of IVs. If the P value of MR-Egger’s intercept is less than 0.05, it indicates that there is significant horizontal pleiotropy of genetic variation.

Thirdly, leave-one-out test: The MR results of the remaining IVs were calculated by excluding individual SNPS one by one to assess whether the SNP affected the association between AA and metabolic reprogramming. If there was a large difference between the MR Effect estimates and the total effect estimates after excluding an instrumental variable, it indicated that the MR Effect estimates were sensitive to that SNP. This analysis helps identify potential outlier SNPs, thereby enhancing the robustness of the conclusions.

Fourthly, directionality test (Steiger Test): The Steiger directionality test was used to calculate the explanatory power of exposure for the outcome (R²_exposure) and the explanatory power of the outcome for exposure (R²_outcome). If R²_exposure > R²_outcome, it indicates that the causal direction is “exposure → outcome,” verifying that the direction of the MR assumption is correct.

### 2.7 SMR analysis

SMR (Summary-Data-Based Mendelian Randomization) (13), using GWAS summary data from GWAS and expression quantitative trait locus (QTL) studies to test for pleiotropic associations between base protein expression levels and complex traits of interest. We download SMR Linux version (1.3.1) from SMR website (https://yanglab.westlake.edu.cn/software/smr), in accordance with the default parameters for performing SMR analysis.

### 2.8 Colocalization analysis

For genes with significant Mendelian randomization associations in eQTLGen, we used the coloc package for colocalization analysis (14). Bayesian approach was used to determine whether the association between gene expression and AA was driven by shared causal variants at specific loci rather than linkage disequilibrium. The colocalization analysis has five assumptions, namely: H0, SNPS within the colocalization region are not associated with either trait. H1, SNPS within the colocalization region are associated with the first trait but not with the second trait. H2, SNPS within the colocalizing region are associated with the second trait but not with the first. H3, SNPS within the colocalizing region were associated with both traits but not with the same locus. H4, SNPS within the co-localized region were associated with both traits and located at the same locus. We considered H4 ≥ 0.8 as a high level of colocalization, and 0.5≤H4≤ 0.8 as a moderate level of colocalization (15).

### 2.9 Full phenomenon association analysis

To evaluate the pleiotropic effects and possible adverse effects of potential therapeutic targets. We analyzed genes related to metabolic reprogramming based on AstraZeneca PheWAS Portal (https://azphewas.com/) and PheWeb Database (https://pheweb.org/). The original study included approximately 15,500 binary and 1500 continuous phenotypes, with individuals in exome-sequencing subgroups obtained from the UK Biobank. These data provide us with a broad perspective, allowing us to comprehensively analyze the associations between different phenotypes.

### 2.10 Immunofluorescence staining

Skin sections were fixed with 4% paraformaldehyde in PBS, permeabilized with 0.01% T Triton X-100 in PBS for 1 h. Blocked tissue was incubated with primary antibody anti-SQSTM1 (abcam, UK, 1:1200) or anti-LC3B (Cell Signaling Technology, USA, 1:2000) overnight at 4°C. After washing, sections were incubated with secondary antibody for 1 hour at room temperature, and nuclei were stained with DAPI (1:10,000). Images were processed on an inverted fluorescent microscope (ZEISS Axio Observer. A1).

### 2.11 Quantitative real-time PCR

Fresh human scalp tissue samples were flash-frozen in liquid nitrogen, thoroughly pulverized into a fine powder, and total RNA was extracted using Trizol reagent (Invitrogen, USA) according to the instructions of the manufacturer. RNA purity and concentrations were determined using the Nanodrop. Reverse transcription of the RNA into cDNA was performed using the TetrocDNA Synthesis Kit (Bioline‐Meridian Bioscience). Real-time quantitative PCR (RT-qPCR) analyses were performed by using the CFX96 Real Time System (Bio-Rad Laboratories). The mRNA level of GAPDH was used as an internal control. All expressions were calculated using the 2^−ΔΔCt^ method. The primers for RT-qPCR are as follows: SQSTM1 forward: 5’- AGGGAACACAGCAAGCT-3’, SQSTM1 reverse: 5’-GCCAAAGTGTCCATGTTTCA-3’; DLD forward: 5’- TTCCCATTTGCTGCTAACA-3’, DLD reverse: 5’-CTGATAAGGTCGGATGTGC-3’; PSAT1 forward: 5’-ACTTCCTGTCCAAGCCAGTGGA-3’, PSAT1 reverse: 5’-CTGCACCTTGTATTCCAGGACC-3’. GAPDH forward:5’-TGCACCACCAACTGCTTAGC-3’, GAPDH reverse: 5’-GGCATGGACTGTGGTCATGAG-3’.

### 2.12 Statistical methods

All data processing and analysis in this article were based on R software (Version 4.3.0). For comparisons of continuous variables between two groups, statistical significance of normally distributed variables was estimated by independent Student’s T-Test, unless otherwise specified. The Mann-Whitney U Test method (Wilcoxon Rank Sum Test) was used to analyze the differences between the variables that were not normally distributed. Kruskal-Wallis test was used for comparison of three or more groups. Spearman correlation analysis was used to calculate the correlation coefficient between different molecules. All statistical *P values* were two-sided if not specified, and a *P value* of less than 0.05 was considered to indicate statistical significance.

## 3 Results

### 3.1 Analysis framework and flow chart

**Figure 1:**
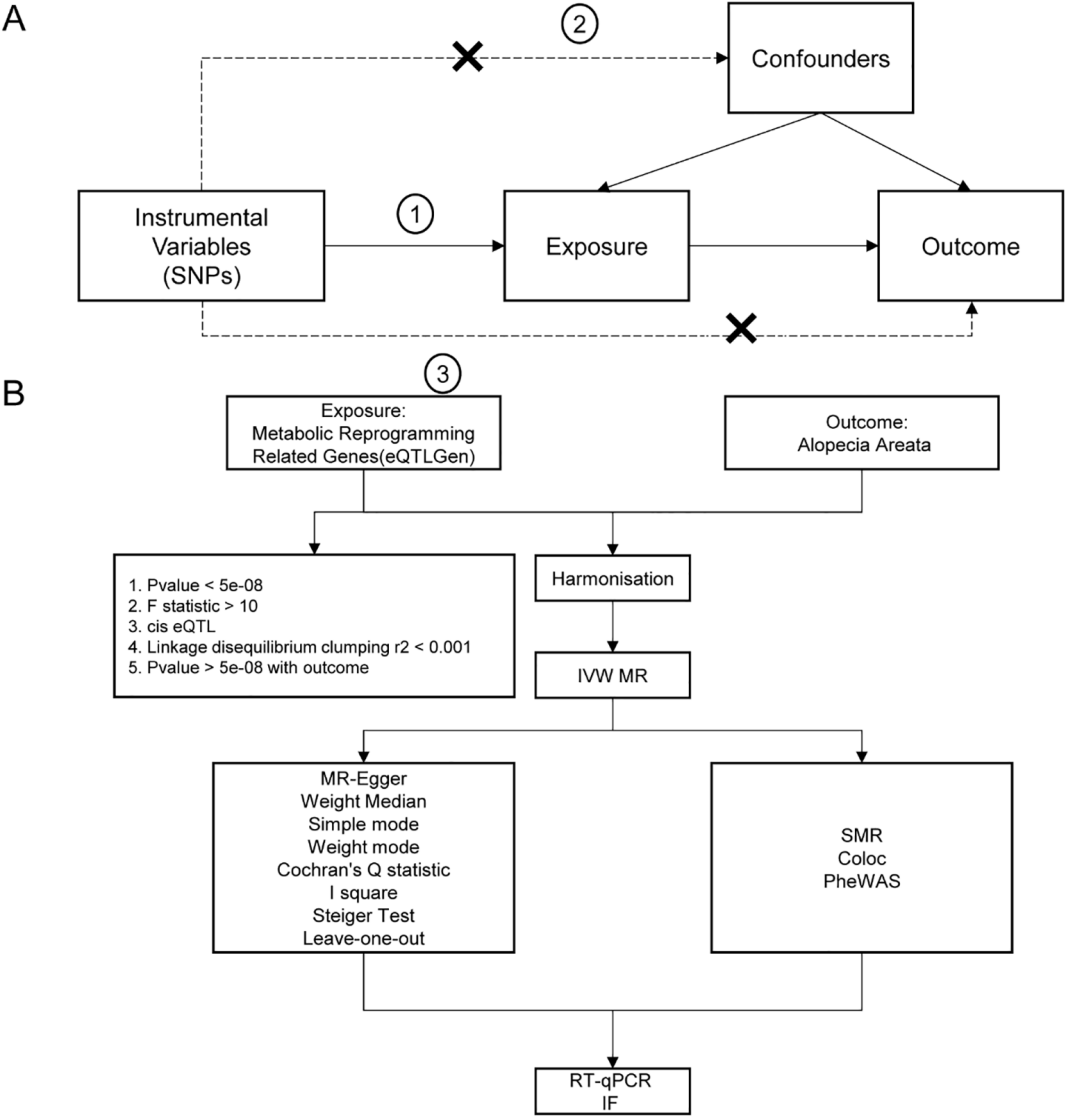
Flow chart of Mendelian randomization analysis. **(A)** The basic assumptions of Mendelian randomization analysis, including, firstly, the association assumption, that is, the selected instrumental variables must be significantly related to the exposure factor; secondly, exclusivity limitation, that is, the instrumental variables could only affect the outcome through the path of “instrumental variable → exposure → outcome”; And thirdly, independence assumption, that is, the instrumental variable must not be significantly related to potential confounders that might affect the exposure or outcome. **(B)** Flow chart of analysis methods in this study. SNP, single nucleotide polymorphism; IVW, inverse variance weighted; MR-Egger, Mendelian randomization-Egger; GWAS, genome-wide association study; SMR, Summary-data-based Mendelian Randomization.

The study’s flowchart is illustrated in **Figure 1**.

### 3.2 MR Analysis of metabolic reprogramming related genes

**Figure 2:**
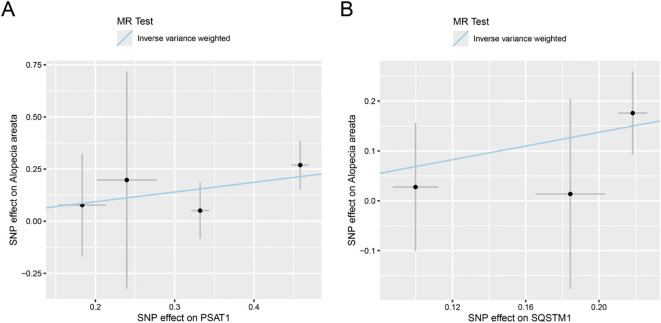
Scatter plot of estimated Mendelian randomization effect of genes on AA. **(A, B)** Scatter plots of Mendelian randomization effect estimate of genes *PSAT1***(A)**, *SQSTM1***(B)** on AA, and the slopes of the lines indicate the magnitude of the predicted causal relationship. MRRGs, Metabolic Reprogramming Related Genes.

We first intersected 96 MRRGs with 19127 genes in eQTLGen to obtain 88 genes (see **Supplementary Table S1** in the appendix). Then we performed two-sample Mendelian randomization (MS) analysis on the cis-eQTL data of these 88 genes and AA by TwoSampleMR. We used *P value* < 0.05 as the screening condition for significant causality to further identify the genes with strong causal association with AA. From the results in **Table 1**, it could be seen that a total of 6 genes (*DLD, NFE2L2, SDHB, SLC2A1, PSAT1, SQSTM1*) have a causal relationship with AA, and the genes *DLD, NFE2L2, SLC2A1, PSAT1, SQSTM1* is positively correlated with the risk of AA. *SDHB* is negatively correlated with the risk of AA. Finally, we plotted the scatter plots of the Mendelian randomization effect estimates of genes *PSAT1* (**Figure 2A**), *SQSTM1* (**Figure 2B**) and AA (plotting genes with Number of SNPs > 1), which showed that the intercept of each model line on the vertical axis was close to 0.

**Table 1:** Mendelian randomization causal effect estimates of genes from eQTLGen for the onset of alopecia areata.

Outcome	Exposure	Method	Number of SNPs	Beta.	Standard error	OR (95% CI)	*P value*
Alopecia Areata	DLD	Wald ratio	1	1.680334741	0.842436049	5.37 (1.03, 27.98)	0.046084995
Alopecia Areata	NFE2L2	Wald ratio	1	3.30115563	1.594716813	27.14 (1.19, 618.19)	0.038446953
Alopecia Areata	SDHB	Wald ratio	1	-1.776883498	0.847322246	0.17 (0.03, 0.89)	0.035988483
Alopecia Areata	SLC2A1	Wald ratio	1	2.885761311	1.009532271	17.92 (2.48, 129.60)	0.004256314
Alopecia Areata	PSAT1	Inverse variance weighted	4	0.46665113	0.212736333	1.59 (1.05, 2.42)	0.028266645
Alopecia Areata	SQSTM1	Inverse variance weighted	3	0.686725813	0.343716918	1.99 (1.01, 3.90)	0.045723155

SNP, single nucleotide polymorphism; OR, odds ratio; CI, confidence interval.

### 3.3 Sensitivity analysis of genes and AA

**Figure 3:**
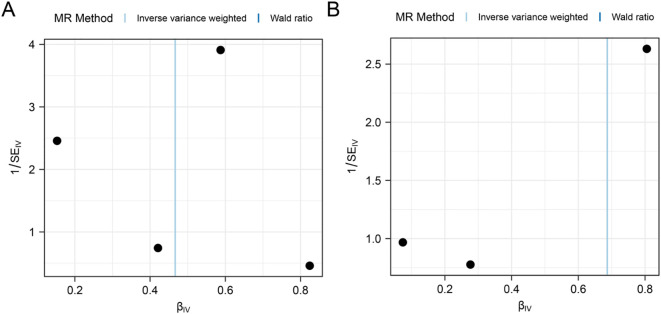
Funnel plot of heterogeneity test for Mendelian randomization analysis of genes for alopecia areata. **(A, B)** Funnel plot of Mendelian randomization heterogeneity test for genes *PSAT1***(A)**, *SQSTM1***(B)** versus alopecia areata (AA).

First, we performed heterogeneity test for significant results (*PSAT1, SQSTM1*) using Cochran Q test and I^2^ statistic, as shown in **Table 2**. The results showed that there was no heterogeneity in the MR Results of genes (*PSAT1, SQSTM1*) for AA (Cochran Q *P value >*0.05), and the heterogeneity ratio was low (I^2^<50%). The funnel plot of the indicator IVs is shown in **Figure 3**, which only shows the results when the number of SNPS is greater than 2, showing that the scatter of the causal association effect is basically symmetrically distributed on both sides of the IVW model line, indicating that there is no potential bias in the results. Indicators with less than 3 SNPS could not be subjected to horizontal pleiotropy test and one-by-one elimination test.

**Table 2:** Heterogeneity test of Mendelian randomization analysis of genes for alopecia areata.

Outcome	Exposure	Method	Q	Q_df	Q_pval	I^2 (%)
Alopecia Areata	PSAT1	MR Egger	0.581212993	2	0.747809886	0
Alopecia Areata	PSAT1	Inverse variance weighted	0.846608672	3	0.838289392	0
Alopecia Areata	SQSTM1	MR Egger	0.348194798	1	0.555136794	0
Alopecia Areata	SQSTM1	Inverse variance weighted	0.550800526	2	0.759268155	0

Q, Cochran’s Q test statistic; Q_df, Q test degree of freedom; Q_pval, *P value* of Q test; The I^2^ statistic reflects the proportion of the heterogeneity part of the instrumental variable in the total variation: if I^2^ ≤ 0, it is set to 0, indicating that no heterogeneity is observed. I^2^ = 0-25%, indicating mild heterogeneity; I^2^ = 25%-50%, indicating moderate heterogeneity; I^2^>50% indicated high heterogeneity. The specific calculation formula is^2^ I= (q-df)/Q×100%.

Then, the MR-Egger regression was used to test the horizontal pleiotropy of IVs. We tested the pleiotropy of genes (*PSAT1, SQSTM1*) and AA (**Table 3**), which showed that genes (*PSAT1, SQSTM1*) were not affected by the horizontal pleiotropy (*P vale*>0.05).

**Table 3:** Genes for alopecia areata Mendelian randomization analysis level pleiotropy test.

Outcome	Exposure	Egger_intercept	Standard error	*P value*
Alopecia Areata	PSAT1	-0.176315772	0.342250617	0.657725228
Alopecia Areata	SQSTM1	-0.110588703	0.245688542	0.730740648

**Figure 4:**
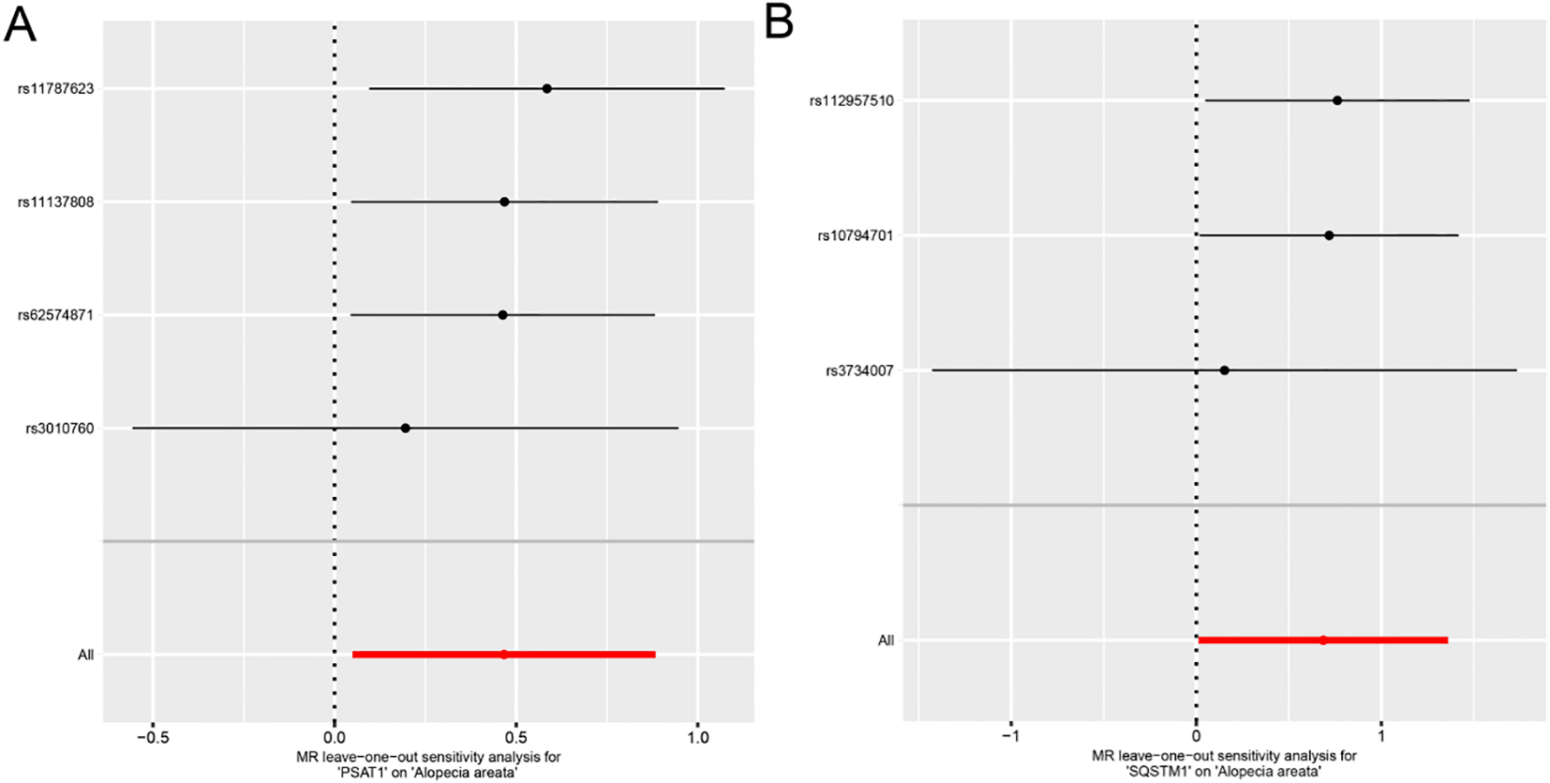
Forest plot of Leave-one-out analysis for Mendelian randomization analysis of genes and alopecia areata. A-B. Forest plots of individual effect estimation results using single SNP locus analysis for genes *PSAT1***(A)**, *SQSTM1***(B)** and alopecia areata (AA).

Each line in the Figure represents the effect size and 95% confidence interval range of the index after the corresponding SNP is removed, and the red line represents the reference effect interval. It could be seen that each line has a high degree of overlap with the red line interval, indicating that the effect estimate will not change significantly with the removal of a single SNP, and the results are stable (**Figure 4**).

To ensure that the causal role of genes in the pathogenesis of AA was in the right direction, we further performed the analysis using steiger’s direction test and found that genes *(DLD, NFE2L2, SDHB, SLC2A1, PSAT1, SQSTM1*) and AA, the *p value* of steiger direction test was much less than 0.05, indicating the correct direction (**Table 4**).

**Table 4:** Mendelian randomization analysis of genes for alopecia areata steiger directionality test.

Exposure	Outcome	Snp_r2.exposure	Snp_r2.outcome	Correct_causal_direction	Steiger_pval
DLD	Alopecia Areata	0.003893237	1.88E-05	TRUE	7.53E-22
NFE2L2	Alopecia Areata	0.001525483	2.03E-05	TRUE	9.50E-09
SDHB	Alopecia Areata	0.007399673	2.08E-05	TRUE	1.68E-17
SLC2A1	Alopecia Areata	0.003329276	3.86E-05	TRUE	1.45E-17
PSAT1	Alopecia Areata	0.064164354	2.68E-05	TRUE	0.00E+00
SQSTM1	Alopecia Areata	0.029250555	2.15E-05	TRUE	1.02E-168

SNP, single nucleotide polymorphism; r^2^, variance explained rate.

### 3.4 SMR analysis and colocalization analysis

**Figure 5:**
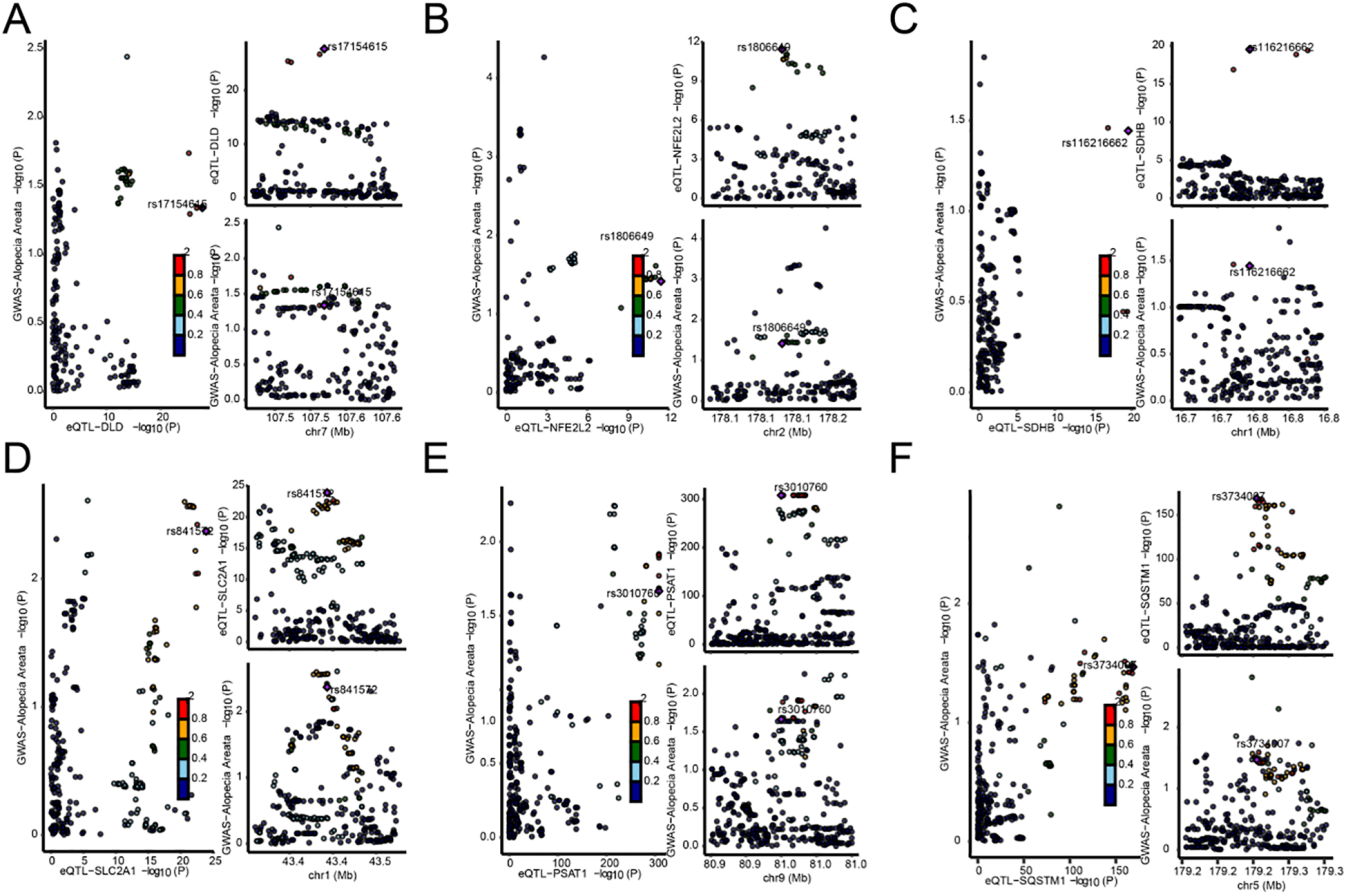
Map of the co-localization region between genes and AA. **(A–F)**. Map of co-localized regions of genes with AA, *DLD***(A)**, *NFE2L2***(B)**, *SDHB***(C)**, *SLC2A1***(D)**, *PSAT1***(E)**, *SQSTM1***(F)**. The left panel of each plot represents the distribution of single nucleotide polymorphisms (SNPS) in genome-wide association studies (GWAS) and expression quantitative Trait loci (eQTL) −log10 (p), with smaller *p values* above the Y-axis. The two plots on the right show the distribution of EQTls and GWAS by sublist, respectively (abscissions are loci for SNPS, and ordinate represent −log10 (p) values for SNPS in that GWAS/eQTL data).

We found six genes (*DLD, NFE2L2, SDHB, SLC2A1, PSAT1, SQSTM1*) with p_SMR < 0.05 (**Table 5**), which further proved that causal relationship existed between the genes (*DLD, NFE2L2, SDHB, SLC2A1, PSAT1, SQSTM1*) and AA. We further performed colocalization analysis at the SNP level to assess evidence for common causal variants between six genes (*DLD, NFE2L2, SDHB, SLC2A1, PSAT1, SQSTM1*) and AA. We ranked SNPS located ±50 kb between genes (*DLD, NFE2L2, SDHB, SLC2A1, PSAT1, SQSTM1*) and AA risk using Bayesian colocalization analysis. Colocalization analysis showed no direct shared causal variants (PP.H4.abf < 0.5) between genes (*DLD, NFE2L2, SDHB, PSAT1, SQSTM1*) expression and AA risk, suggesting that other traits might mediate the causal relationship. A direct shared causal variant (PP.H4.abf > 0.5) was found between gene (*SLC2A1*) expression and AA risk. Finally, we used the R package locus compare to display the genes *DLD* (SNP: rs17154615, **Figure 5A**), *NFE2L2* (SNP: rs1806649, **Figure 5B**), *SDHB* (SNP: rs116216662, **Figure 5C**), *SLC2A1* (SNP: rs841572, **Figure 5D**), *PSAT1* (SNP: rs3010760, **Figure 5E**), *SQSTM1* (SNP: rs3734007, **Figure 5F**) and distribution of major SNPS in AA (**Figures 5A–F**, **Table 6**).

**Table 5:** Results of SMR analysis of genes for alopecia areata.

Gene	Exposure	b_SMR	se_SMR	p_SMR
ENSG00000091140	DLD	1.68033	0.856026	0.04965303
ENSG00000116044	NFE2L2	3.30115	1.66386	0.0472522
ENSG00000117118	SDHB	-1.77688	0.868944	0.04086704
ENSG00000117394	SLC2A1	2.88576	1.0481	0.005899334
ENSG00000135069	PSAT1	0.587389	0.25612	0.02182403
ENSG00000161011	SQSTM1	0.805355	0.381122	0.034591

SMR, Summary-data-based Mendelian Randomization; SNP, single nucleotide polymorphism.

**Table 6:** Results of colocalization analysis of genes and alopecia areata coloc.

Exposure	nsnps	PP.H0.abf	PP.H1.abf	PP.H2.abf	PP.H3.abf	PP.H4.abf
SDHB	279	9.82236E-13	0.878570295	2.45176E-14	0.022	0.100
SLC2A1	336	1.26082E-18	0.442031391	8.20274E-20	0.028	0.530
NFE2L2	215	1.53388E-06	0.723219345	1.77396E-07	0.083	0.193
SQSTM1	317	3.8241E-162	0.770763911	1.5225E-163	0.030	0.199
DLD	226	3.69145E-22	0.797845111	1.14519E-23	0.025	0.178
PSAT1	399	0	0.818538943	0	0.039	0.142

### 3.5 Gene whole-phenomenon association analysis

Using PheWAS Portal and PheWeb databases, we performed full phenomenon Mendelian randomization to determine possible side effects of targeting *DLD, NFE2L2, SDHB, SLC2A1, PSAT1, SQSTM1.* It was found that, as shown in the PheWeb database (**Table 7**), The gene *DLD* is associated with Cholelithiasis and cholecystitis, Cholelithiasis, Inflammatory bowel disease and gastroenteritis and colitis other gastroenteritis and colitis), Ulcerative colitis, and the *NFE2L2* gene with Abnormal sputum *p value* < 5 × 10^–8^), Hemoptysis (*p value* < 5 × 10^–8^) at the genome-wide significance level. The PheWAS Portal (**Table 8**) showed that except for the significant relationship between the gene *SDHB* and Chapter II Neoplasms at the genome-wide significance level (*p value* < 5 × 10^–8^), All other genes showed no significant relationship at the genome-wide significance level (*p value >*5 × 10^–8^). Meanwhile, no association was reported between Abnormal sputum, Hemoptysis and AA.

**Table 7:** Results of gene-wide phenomenon association analysis (PheWeb).

Gene	Top *P value* in gene	Phenotype
DLD	3.70E-12	Cholelithiasis and cholecystitis
DLD	2.00E-11	Cholelithiasis
DLD	2.80E-09	Inflammatory bowel disease and other gastroenteritis and colitis
DLD	3.20E-09	Ulcerative colitis
DLD	5.10E-08	Transient mental disorders due to conditions classified elsewhere
DLD	8.60E-08	Abnormal heart sounds
NFE2L2	1.70E-08	Abnormal sputum
NFE2L2	4.00E-08	Hemoptysis
NFE2L2	7.60E-08	Folate-deficiency anemia
NFE2L2	3.80E-07	Angina pectoris
NFE2L2	4.80E-07	Benign neoplasm of brain, cranial nerves, meninges
NFE2L2	6.60E-07	Benign neoplasm of brain and other parts of nervous system
SDHB	8.50E-08	Type 1 diabetes with ophthalmic manifestations
SDHB	1.80E-07	Symptoms involving respiratory system and other chest symptoms
SDHB	3.50E-07	Abnormal findings on exam of gastrointestinal tract/abdominal area
SDHB	5.80E-07	Sexually transmitted infections (not HIV or hepatitis)
SDHB	8.00E-07	Bipolar
SDHB	8.40E-07	Abnormal function study of cardiovascular system
SLC2A1	1.70E-07	Other conditions of brain
SLC2A1	5.60E-07	Cardiogenic shock
SLC2A1	8.30E-07	Benign neoplasm of bone and articular cartilage
SLC2A1	1.00E-06	Cyst and pseudocyst of pancreas
PSAT1	1.90E-07	Hepatitis NOS
PSAT1	3.70E-07	Poisoning by antibiotics
PSAT1	3.80E-07	Nerve plexus lesions
PSAT1	5.20E-07	Encephalopathy, not elsewhere classified
PSAT1	7.70E-07	Excessive or frequent menstruation
PSAT1	9.90E-07	Leukoplakia of oral mucosa
SQSTM1	1.60E-07	Ptosis of eyelid
SQSTM1	3.90E-07	Aneurysm of iliac artery
SQSTM1	4.40E-07	Sqstm1 Other conditions or status of the mother complicating pregnancy, childbirth, or the puerperium
SQSTM1	4.90E-07	Acute pharyngitis
SQSTM1	4.90E-07	Acute bronchitis and bronchiolitis
SQSTM1	6.40E-07	Other CNS infection and poliomyelitis

**Table 8:** Results of gene whole-phenomenon association analysis (PheWAS Portal).

Gene	Top *P value* in gene	Phenotype
DLD	0.0000206	Chapter XI Diseases of the digestive system
DLD	0.0000303	Chapter XI Diseases of the digestive system
DLD	0.0000567	Chapter XI Diseases of the digestive system
DLD	0.00008903	Chapter II Neoplasms
DLD	0.00009082	Chapter XI Diseases of the digestive system
NFE2L2	0.00002348	Chapter XIII Diseases of the musculoskeletal system and connective tissue
NFE2L2	0.00005559	Chapter V Mental and behavioral disorders
NFE2L2	0.00007251	Chapter II Neoplasms
NFE2L2	0.00007251	Chapter II Neoplasms
NFE2L2	0.0000762	Chapter V Mental and behavioral disorders
SDHB	1.10E-10	Chapter II Neoplasms
SDHB	3.62E-10	Chapter II Neoplasms
SDHB	2.70E-09	Chapter II Neoplasms
SDHB	9.69E-09	Chapter II Neoplasms
SDHB	1.75E-08	Chapter II Neoplasms
SLC2A1	0.0000774	Chapter IX Diseases of the circulatory system
SLC2A1	0.0001737	Chapter XIII Diseases of the musculoskeletal system and connective tissue
SLC2A1	0.0002443	Chapter II Neoplasms
SLC2A1	0.000262	Chapter IX Diseases of the circulatory system
SLC2A1	0.0002694	Chapter II Neoplasms
PSAT1	0.00001421	Chapter X Diseases of the respiratory system
PSAT1	0.00002441	Chapter XIV Diseases of the genitourinary system
PSAT1	0.00002827	Chapter X Diseases of the respiratory system
PSAT1	0.00004562	Chapter X Diseases of the respiratory system
PSAT1	0.0000518	Chapter X Diseases of the respiratory system
SQSTM1	4.76E-07	Chapter XIII Diseases of the musculoskeletal system and connective tissue
SQSTM1	0.000001454	Chapter XIII Diseases of the musculoskeletal system and connective tissue
SQSTM1	0.000001757	Chapter XIII Diseases of the musculoskeletal system and connective tissue
SQSTM1	0.000001874	Chapter XIII Diseases of the musculoskeletal system and connective tissue
SQSTM1	0.000002282	Chapter XIII Diseases of the musculoskeletal system and connective tissue

### 3.6 Dysregulated expression of SQSTM1 and blocked autophagy activity in AA

**Figure 6:**
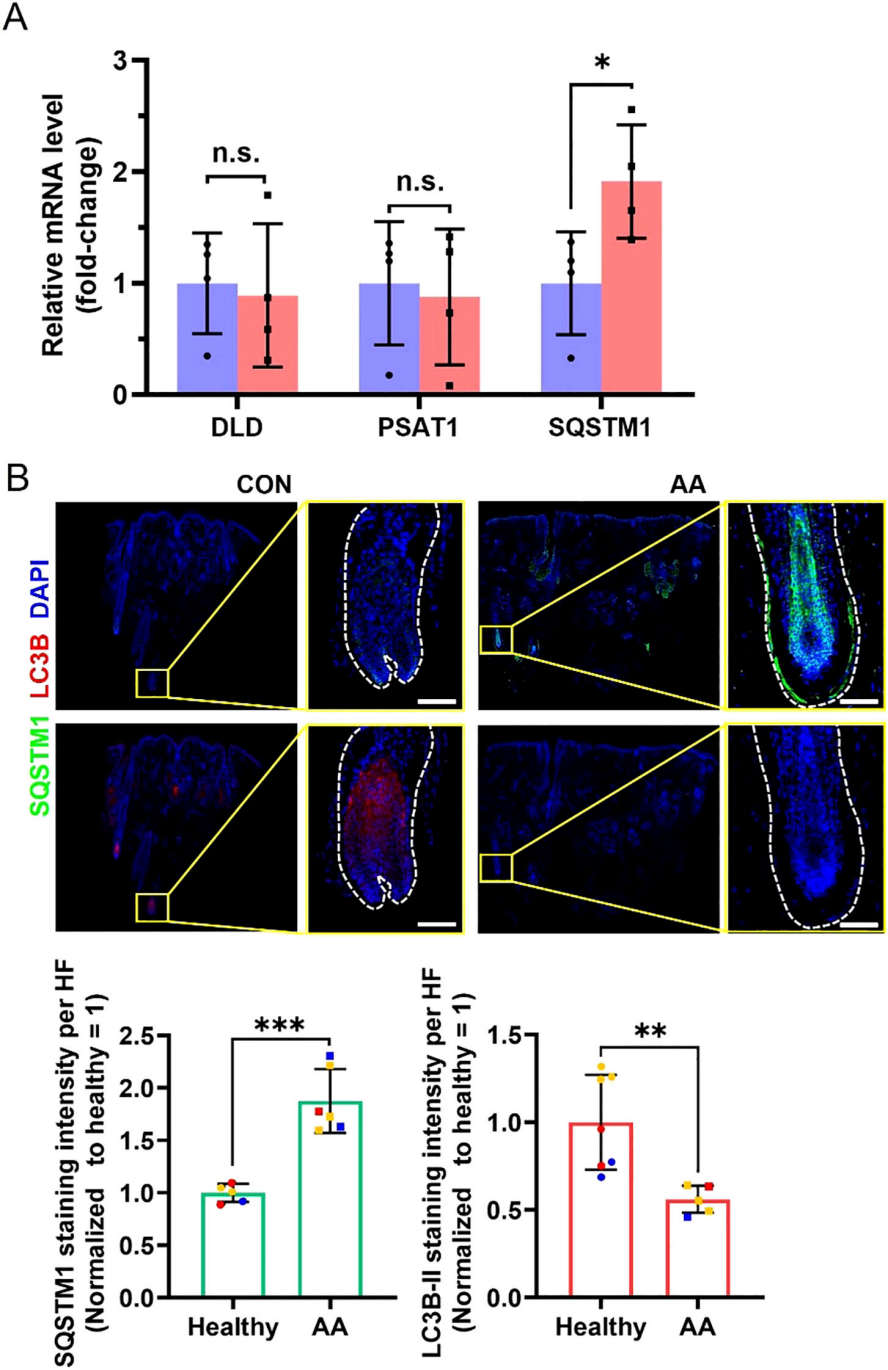
Differences in related mRNA and protein levels in scalp biopsies between healthy controls and AA patients. **(A)** RT-qPCR analyses for relative mRNA level of *DLD*, *PSAT1* and *SQSTM1*. N = 4 independent samples. **(B)** Representative immunofluorescent images of SQSTM1 and LC3B in healthy controls and AA patients. Scale bars, 50 μm. Bottom, quantification of these two proteins’ expression in the hair follicle region. Multiple hair follicle fields included in the statistical analysis were derived from 3 independent samples. All data are mean ± s.d. *p < 0.05, **p < 0.01, *** p < 0.001, ns: non-significant.

Furthermore, we sought to investigate whether the potential risk genes significantly associated with AA exhibit altered expression during disease progression. Scalp biopsies from AA patients (confirmed by histopathology) were obtained, and we quantitatively measured the mRNA expression levels of DLD, PSAT1, and SQSTM1 by RT-qPCR. The results demonstrated that while DLD and PSAT1 mRNA levels in lesional areas showed no significant difference compared to healthy controls, SQSTM1 mRNA level was markedly elevated in AA patients (**Figure 6A**). Given that SQSTM1 serves as a crucial indicator of autophagic activity, we subsequently evaluated autophagy levels in hair follicles through immunofluorescence staining (**Figure 6B**). Quantitative analysis revealed that compared to healthy controls, AA-affected hair follicles exhibited higher SQSTM1 expression but lower LC3B-II expression, consistent with a state of autophagy suppression. It should be noted that the LC3B (D11) XP Rabbit mAb (Cell Signaling Technology, #3638) used for LC3B immunostaining exhibits stronger reactivity with endogenous LC3B-II (16). These results indicate that while the synthesis of SQSTM1, one of key autophagy-related protein is increased in lesional areas of AA patients, autophagic flux is significantly blocked.

## 4 Discussion

AA is currently recognized as an autoimmune and inflammatory disease, affecting approximately 2% of the population, with potential antigens including melanocytes and their products, as well as keratinocytes. The disease is predominantly mediated by CD8+ T cells. Clinically, AA is often accompanied with type II inflammatory diseases and other autoimmune diseases. Psychological stress and Infection induction are known triggers for AA, however, some severe AA usually has no obvious predisposing factors (3). Despite significant progress in the field, there remains a notable gap in our knowledge regarding the precise genetic mechanisms underlying AA. While numerous studies have identified candidate genes associated with the disease, the complexity of gene interactions and their effects on metabolic pathways require further elucidation. Emerging evidence may suggest a potential link between AA and metabolic reprogramming. Firstly, immune dysregulation in AA might influence cellular metabolic pathways, including energy and lipid metabolism, which in turn could affect immune cell function. Secondly, psychological stress, a known trigger for AA, could induce metabolic reprogramming. Thirdly, metabolic reprogramming is often accompanied by inflammatory responses (4, 5), and the accumulation of inflammatory cells around hair follicles in AA might be related to altered metabolic states. Lastly, metabolic reprogramming might lead to nutrient deficiencies, adversely affecting hair growth and health. This study aims to elucidate the genetic factors contributing to AA by employing a comprehensive MR analysis. The analytical framework of this study is built upon the MR-Base platform proposed by Hemani et al. (11), and the SMR method developed by Zhu et al. (13) and Wu et al. (17) Our findings reveal six MRRGs—*DLD, NFE2L2, SDHB, SLC2A1, PSAT1*, and *SQSTM1*—that demonstrate significant causal associations with AA. Among these, *DLD, NFE2L2, SLC2A1, PSAT1, and SQSTM1* were positively correlated with the disease, while *SDHB* exhibited a negative correlation.

Notably, we found that AA-affected hair follicles exhibited higher SQSTM1 expression but lower LC3B-II expression, consistent with a state of autophagy suppression. SQSTM1 functions as a critical signaling node that coordinates both mTORC1 activation on lysosomes and Keap1-Nrf2 pathway regulation on autophagic cargoes, while simultaneously serving as an essential adaptor protein for selective autophagy processes (18–20). Abnormalities in autophagy are frequently associated with various diseases, such as Crohn disease, celiac disease, multiple sclerosis, systemic lupus erythematosus, and type 1 diabetes (22, 23). Recent studies have elucidated the link between autophagic dysregulation and mitochondrial dysfunction in driving neurodegeneration (24).

Autophagy plays a direct regulatory role in hair regeneration, where its activation promotes hair growth in telogen hair follicles (25). Studies have indicated that an imbalance between mitophagy and oxidative stress—leading to hair follicle damage—plays a significant role in androgenetic alopecia (26, 34). Previous GWAS analysis has linked two autophagy-related pathways involving PARK2 and PFKFB3 with increased susceptibility to AA (27), also revealed that variations in genes such as STX17, CLEC16A, and BCL2L11/BIM, which play a role in regulating autophagy, are predisposing genetic loci for AA (28, 29). In addition, identified multiple AA patients with copy number variations (CNV) in the genomic region spanning ATG4B, which is a cysteine protease involved in posttranslational modification of the autophagy protein LC3 (22, 30–32). In fact, impairment in autophagy has been implicated in the loss of immune tolerance in human AA (21). Recent studies have elucidated that *Centella asiatica* (CAW) can induce changes in the DNA methylation pattern of peripheral blood in aged mice associated with increased lifespan and processes associated with healthy aging, thereby regulating immune-related metabolic pathways (33). In AA, the relationship between changes in autophagy and epigenetic modifications merits investigation.

In our research, autophagic activity in hair follicles of AA is suppressed, while SQSTM1 mRNA levels are significantly increased compared to healthy controls. This observation suggests that the elevated SQSTM1 levels result not only from impaired autophagy-mediated degradation (leading to SQSTM1 accumulation), but also potentially from active upregulation of SQSTM1 synthesis. Generally, autophagy plays a protective role in cells, but disruption of autophagy mechanisms or excessive autophagic flux usually leads to cell death which propagates aberrant immune responses (34) and may trigger metabolic reprogramming. From the perspective of metabolic reprogramming, we propose a hypothesis that abnormal SQSTM1 gene expression in AA patients may lead to dysfunctional SQSTM1 protein biology, thereby impairing autophagy. In response, the system attempts to compensate by increasing SQSTM1 synthesis, yet this fails to restore autophagic flux. SQSTM1 transcriptional activation may result from Nrf2-mediated transcription activation, and subsequently triggers NF-κB activation and inflammatory response (35). We postulate that increased SQSTM1 transcription and protein accumulation in lesional follicles predict increased susceptibility to inflammation response, and autophagic impairment, driving AA pathogenesis.

However, this study still has limitations and requires further improvement. First, the Mendelian randomization analysis conducted relies on data from public databases, with study samples predominantly sourced from European populations, which may introduce biases related to population applicability. Second, the Alopecia Areata GWAS dataset utilized in this study contains a relatively small number of case samples (289 cases), which could compromise statistical power. Third, the eQTLGen database is primarily based on peripheral blood samples, and its gene expression profiles may differ from those of local hair follicle tissues, thereby restricting the tissue-specific interpretation of the results to some extent. Furthermore, while Mendelian randomization can mitigate confounding biases, it may still be susceptible to the effects of horizontal pleiotropy and the accuracy of instrumental variables. Finally, the sample sizes for RT-qPCR and immunofluorescence validation are limited; future studies should expand the sample sizes and integrate functional experiments to further validate the mechanisms of action of these key genes. Moreover, we will further validate the functional role of SQSTM1 in the pathogenesis of AA through cellular and animal model experiments (e.g., mechanisms of autophagic flux and immune regulation), and enhance prospects for clinical translation.

In conclusion, this research elucidates association between metabolic reprogramming and AA, particularly highlighting the role of SQSTM1, advancing our understanding of AA pathophysiology and is a promising therapeutic target.

## Data Availability

The original contributions presented in the study are included in the article/**Supplementary Material**. Further inquiries can be directed to the corresponding author.

## Glossary

AA: Alopecia areata
ATG4B: Autophagy Related 4B Cysteine Peptidase
BIM: BCL2 Interacting Mediator of Cell Death
CAW: Centella asiatica Water extract
cDNA: Complementary deoxyribonucleic acid
CFX96: CFX96 Real Time System
CLEC16A: C-Type Lectin Domain Family 16 Member A
CNV: Copy Number Variation
DAPI: 4’,6-Diamidino-2-phenylindole
FDR: False Discovery Rate
GWAS: Genome-Wide Association Study
IVs: Instrumental Variables
IVW: Inverse Variance Weighted
JAKi: Janus Kinase Inhibitors
Keap1: Kelch-Like ECH-Associated Protein 1
LD: Linkage Disequilibrium
LC3B: Microtubule-Associated Protein 1 Light Chain 3 Beta
MAF: Minor Allele Frequency
mTORC1: Mechanistic Target of Rapamycin Complex 1
MR: Mendelian Randomization
MR-Egger: Mendelian Randomization-Egger
MRRGs: Metabolic Reprogramming-Related Genes
NF-κB: Nuclear Factor Kappa-Light-Chain-Enhancer of Activated B Cells
Nrf2: Nuclear Factor Erythroid 2-Related Factor 2
PARK2: Parkin RBR E3 Ubiquitin Protein Ligase
PBMCs: Peripheral Blood Mononuclear Cells
PBS: Phosphate-Buffered Saline
PFKFB3: 6-Phosphofructo-2-Kinase/Fructose-2,6-Bisphosphatase 3
PheWAS: Phenome-Wide Association Study
PP.H4.abf: Posterior Probability of Hypothesis 4 (colocalization) using Approximate Bayes Factor
PSAT1: Phosphoserine Aminotransferase 1
qPCR: Quantitative Polymerase Chain Reaction
RT-qPCR: Quantitative Real-Time Polymerase Chain Reaction
SNP: Single Nucleotide Polymorphism
SMR: Summary-Data-Based Mendelian Randomization
SQSTM1: Sequestosome 1
STX17: Syntaxin 17
T-Test: Student’s T-Test
U Test: Mann-Whitney U Test.
