# Supplementary material for: Association between SQSTM1 dysregulation and risk in alopecia areata: a Mendelian randomization study
Source: Front Immunol. 2025 Nov 25;16:1652444. doi: 10.3389/fimmu.2025.1652444 (PMC12685797; doi:10.3389/fimmu.2025.1652444)
Supplement: Supplementary file 1 [file Table1.docx]

**Table S1 List of 88 MRRGs in eQTLGen**

| MRRGs | | | | |
| --- | --- | --- | --- | --- |
| GFPT1 | BCAT2 | PSPH | TALDO1 | KPNA2 |
| TKT | GLS | SLC7A5 | DLST | RPE |
| PFKP | SQSTM1 | SLC1A5 | DLD | NFE2L2 |
| UCP2 | BCKDHA | SDHB | ME1 | IDH3A |
| PSAT1 | MTOR | PGM3 | SLC43A1 | GATA3 |
| PPARGC1A | RPIA | IDH1 | SLC2A1 | MDH2 |
| HK2 | STK11 | IDH2 | PRKAA1 | BRAF |
| SUCLG2 | CARM1 | ALDOB | HK3 | DLAT |
| SOAT1 | PFKL | HIF1A | SHMT2 | GPT |
| SLC16A3 | ENO1 | SLC16A1 | TP53 | TGFB1 |
| FOXK1 | FAM210B | HMGCR | GLUD1 | ACO2 |
| PDHB | PDK4 | SLC16A4 | MYC | TUG1 |
| GAPDH | UAP1 | GART | KRAS | GNPNAT1 |
| PYCR2 | PDHX | GPI | FOXK2 | PKM |
| FASN | LDHA | MDH1 | PGD | PYCR1 |
| PDK1 | HK1 | GOT1 | GLS2 | FH |
| GOT2 | PDCD1 | PPAT | LDLR | CS |
| PGAM1 | ACLY | PAICS |  |  |

MRRGs，Metabolic Reprogramming Related Genes。
